# Heat Transfer Analysis of MHD Thin Film Flow of an Unsteady Second Grade Fluid Past a Vertical Oscillating Belt

**DOI:** 10.1371/journal.pone.0103843

**Published:** 2014-11-10

**Authors:** Taza Gul, Saeed Islam, Rehan Ali Shah, Ilyas Khan, Asma Khalid, Sharidan Shafie

**Affiliations:** 1 Department of Mathematics, Abdul Wali Khan University, Mardan Khyber Pakhtunkhwa, Pakistan; 2 Department of Mathematics, University of Engineering and Technology, Peshawar Khyber Pakhtunkhwa, Pakistan; 3 Department of Basic Sciences, College of Engineering Majmaah University, Majmaah, Saudi Arabia; 4 Department of mathematical Sciences, Faculty of science, University Teknology Malaysia, UTM Johor Bahru, Johor, Malaysia; Bascom Palmer Eye Institute, University of Miami School of Medicine; United States of America

## Abstract

This article aims to study the thin film layer flowing on a vertical oscillating belt. The flow is considered to satisfy the constitutive equation of unsteady second grade fluid. The governing equation for velocity and temperature fields with subjected initial and boundary conditions are solved by two analytical techniques namely Adomian Decomposition Method (ADM) and Optimal Homotopy Asymptotic Method (OHAM). The comparisons of ADM and OHAM solutions for velocity and temperature fields are shown numerically and graphically for both the lift and drainage problems. It is found that both these solutions are identical. In order to understand the physical behavior of the embedded parameters such as Stock number, frequency parameter, magnetic parameter, Brinkman number and Prandtl number, the analytical results are plotted graphically and discussed.

## Introduction

Thin-film flow is significant regarding broad class of physical applications and attracts the attention of physicists, engineers and chemists. In the field of chemical engineering, thin film layers are functioning to design efficient and gainful development units such as thin-film reactors, evaporators, condensers, distillation columns and heat exchangers. The enormous benefit of thin film layers is related to their tiny thickness which, in turn, results in large heat- and mass-transfer areas per unit volume. Further, thin fluid layers have been executed in circumstances where a film of fluid layers is over a solid surface such as in different coating processes [1]. At the micron scale, thin layer is of particular importance, specified by a large scale of microfluidic devices, as evaluated in the work of Stone et al. [2] and Squires and Quake [3].

In physical, chemical and biological sciences, thin film flows have been used in micro-channel heat sinks to provide cooling for nanotechnologies. In environmental and geophysical engineering, thin film flows have been related with geological problems such as lava, debris flows and mudslides [4], [5];

Keeping in view the rich applications of non-Newtonian fluids in engineering and industry, such fluids have been widely studied. Ample research has been carried out in this field. Considerable efforts have been made to study non-Newtonian fluids through analytical and numerical treatment.

One of the well-known model amongst non-Newtonian fluids is the class of second grade fluids which has its constitutive equations based on strong theoretical foundations. Some development and relevant work on this topic is the wire coating in a straight annular die for unsteady second grade fluid discussed by Rehan et al. in [6].

They modeled the unsteady second grade fluid flow between wire and die with one oscillating boundary and the other stationary in the form of partial differential equation. Similar results can also be found in [7], [9]. On the other hand, Samiulhaq et al. [10] investigated unsteady free convection flow of a second grade fluid. They have compared the influence of ramped temperature and isothermal temperature on the velocity field and skin friction through different cases in the presence of magnetic field as well as porosity. Ali et al. [11] studied the closed form solutions for unsteady second grade fluid near vertical oscillating plate. They have shown the effect of various physical parameters on the velocity and temperature fields.

The physical importance of thin film has been researched and discussed by several authors. For examples, thin film flow of a power law model liquid falling an inclined plate was discussed by Miladinova et al. [12], wherein they observed that saturation of non-linear interaction occurred in a finite amplitude permanent wave. Alam et al. [13] investigated the thin-film flow of Johnson-Segalman fluids for lifting and drainage problems. They observed the effect of various parameters on the lift and drainage velocity profiles. To solve real world problems, several approximate techniques have been used in mathematics, fluid mechanics and engineering sciences. Some of the common methods are, HAM and OHAM [14], [15]. Application of optimal Homotopy asymptotic method for solving non-linear equations arising in heat transfer was investigated by Marinca and Herisanu [16]. They have also discussed an optimal Homotopy asymptotic method applied to steady flow of a fourth-grade fluid past a porous plate [17]. These methods deal with the nonlinear problems effectively. Mabood et al. [18] discussed OHAM solution of viscoelastic fluid in axisymmetric heated channels. They have shown that the results of OHAM are comparatively better than other methods' results. Some development in this direction is discussed in [19]–[27]. Taza Gul et al. [28] investigated effects of MHD on thin film flow of third grade fluids for lifting and drainage problems under the action of heat dependent viscosity. The effects of various parameters on the lift and drainage velocity profiles are also studied.

The main objective of this work is to study the effects of oscillation into a MHD thin film flow of an unsteady second grade fluid on a vertical oscillating belt using ADM and OHAM. In 1992, Adomian [29], [30] introduced the ADM for the approximate solutions for linear and non linear problems. Wazwaz [31], [32] used ADM for the reliable treatment of Bratu-type and Rmden-Fowler equations. In a comparative study, Taza Gul et al. [33] used ADM and OHAM for solution of thin film flow of a third grade fluid on a vertical belt with slip boundary conditions.

The convergence of the decomposition series was cautiously examined by several researchers to verify the fast convergence of the resulting series. Cherruault examined the convergence of Adomian's method in [34]. Cherruault and Adomian presented a new proof of convergence of the method in [35].

## Basic Equations

The constitutive equations governing the problem (equation of continuity, momentum and energy) under the influence of externally imposed transverse magnetic field are:
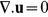
(1)

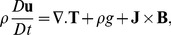
(2)

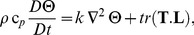
(3)where 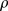, is the constant density, 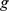 denotes gravity, 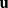 is velocity vector of the fluid, 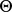 defines temperature, 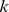 is the thermal conductivity, 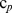 is specific heat, 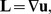 
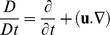
 denotes material time derivative, and 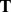 is the Cauchy stress tensor.

One of the body force term corresponding to MHD flow is the Lorentz force 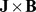. Where 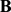 is the total magnetic field and 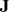 is the current density. By using Ohm's law, the current density is given as 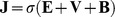



where 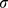 is electrical conductivity of the fluid, 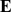 is the electric field, 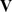 is the velocity vector field, 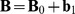 with 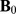 is the imposed magnetic field and 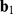 is the induced magnetic field. The current density
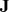
with the assumptions 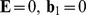 and 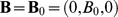, where 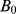 is the strength of applied magnetic field 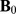, modifies to 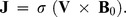 Finally the Lorentz force becomes
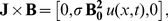
(4)


Cauchy stress tensor 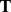 is given by
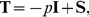
(5)where 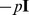 denotes spherical stress and shear stress 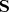, is defined as
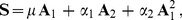
(6)

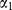
 and 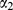 are the material constants and 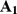,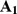 are the kinematical tensors given by
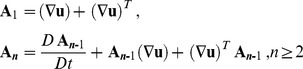
(7)


### Formulation of the Lift Problem

Consider, a wide flat belt moves vertically at time 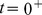, the belt is oscillated and translated with constant speed 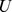 through a large bath of second grade liquid. The belt carries a layer of liquid of constant thickness 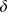. Coordinate system is chosen for analysis in which the y-axis is taken parallel to the belt and x-axis is perpendicular to the belt. Uniform magnetic field is applied transversely to the belt. It has been assumed that the flow is unsteady and laminar after a small distance above the liquid surface layer.

Velocity and temperature fields are defined as:
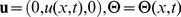
(8)


Oscillating boundary conditions are:
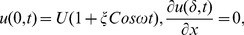
(9)

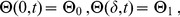
(10)


Here 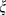 is used as amplitude in [6] and [9]. 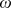 is used as frequency of the oscillating belt.

Inserting the velocity field from Eq.(8) in continuity Eq.(1) and in momentum Eqs.(2) and (4), the continuity Eq.(1) is satisfied identically and momentum Eqs. (2) and (4) are reduced to the following components of stress tensor as: 
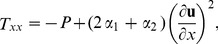
(11)

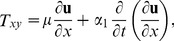
(12)

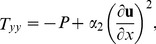
(13)

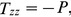
(14)

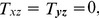
(15)making use of Eqs. (11–15) in Eq.(2,3), the momentum and energy Eqs. (2,3) are reduced to, 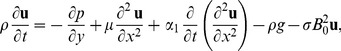
(16)

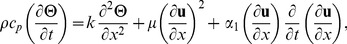
(17)


Introducing the following non-dimensional variables 
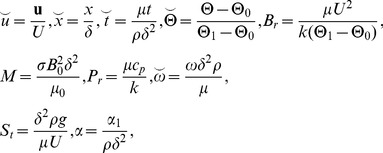
(18)where 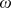 is the frequency parameter, 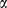 is non-Newtonian effect, 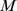 is magnetic parameter, 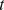 is time parameter, 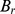 is Brinkman number, 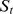 is Stock's number and 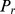 is the Prandtl number.

On inserting the above dimensionless variables in Eqs. (16, 17), when 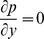, the momentum and energy equations become,
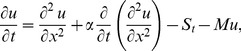
(19)

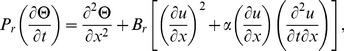
(20)


From Eqs. (9, 10), the non-dimensional boundary conditions are:
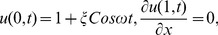
(21)

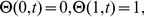
(22)


## Analysis of Adomain Decomposition Method

The Adomian Decomposition Method (ADM) is used to decompose the unknown function 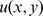 into a sum of an infinite number of components

defined by the decomposition series.
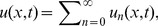
(23)


The decomposition method is used to find the components 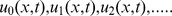 separately. The determination of these components can be obtained through simple integrals.

To give a clear overview of ADM, we consider the linear partial differential equation in an operator form as
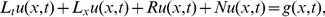
(24)

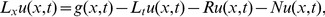
(25)


Where 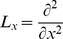 and 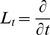 are linear operators in the partial differential equation and are easily invertible, 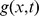 is a source term, 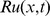 is a remaining linear term and 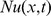 is non-linear analytical term expandable in the Adomian polynomials 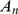


After applying the inverse operator 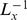 to both sides of Eq. (25).
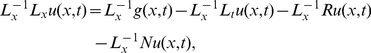
(26)

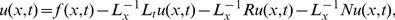
(27)


Here, the function 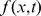 represents the terms arising from 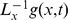 after using the given conditions. 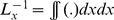 is used as inverse operator for the second order partial differential equation. Similarly, it is used for higher order partial differential equation 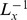 and 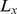 depend on the order of the partial differential equation.

Adomian Decomposition Method defines the series solution 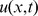 as,
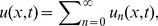
(28)

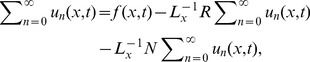
(29)


The non-linear term expanding in Adomian polynomials as,
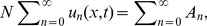
(30)where the components 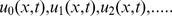 are periodically derived as
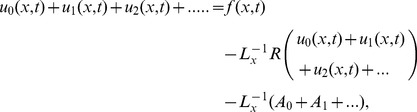
(31)


To determine the series components 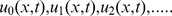 it is important to note that ADM suggests that the function 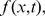 actually described the zeroth component 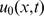, is usually defined by the function 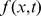 described above.

The formal recursive relation is defined as:
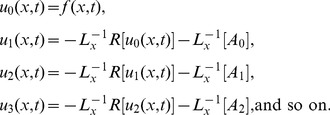
(32)


## Analysis of Optimal Homotopy Asymptotic Method

For the analysis of OHAM, we consider the boundary value problem as
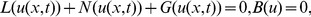
(33)


Where 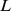 is a linear operator in the differential equation, 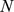 is a non-linear term, 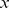 is an independent variable, 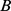 is a boundary operator and 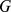 is a source term. According to OHAM, we construct a set of equation. 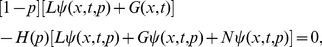
(34)



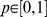
 is an embedding parameter, 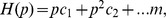 is an auxiliary function and 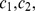 are auxiliary constants and 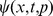 is an unknown function. Obviously, when 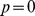 and 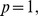 it holds that:
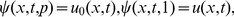
(35)

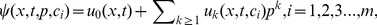
(36)


Inserting Eq.(30) in Eq.(28), assembling the similar powers of 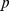 and comparing each coefficient of 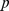 to zero. The partial differential equations are solved with the given boundary conditions to get 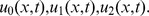


The general solution of Eq.(27) can be written as
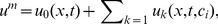
(37)


The coefficients 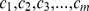 are the functions of 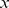.

Inserting Eq. (31) in Eq.(27), the residual is obtained as:
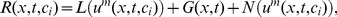
(38)


Numerous methods like Galerkin's Method, Ritz Method, Method of Least Squares and Collocation Method are used to find the optimal values of 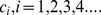 We apply the Method of Least Squares in our problem as given below:
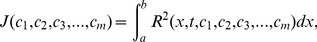
(39)



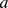
 and 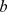 are the constant values taking from domain of the problem.

Auxiliary constants 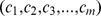 can be obtained from:
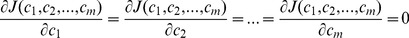
(40)


Finally, from these auxiliary constants, the approximate solution is well-determined.

### The ADM Solution of Lifting Problem

The inverse operator 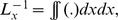 is applied on the second order differential Eq. (16) and is according to the standard form of ADM from Eq.(27):
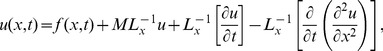
(41)

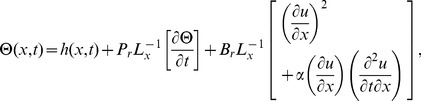
(42)Summation is used for the series solutions of Eqs. (41,42):
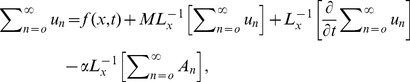
(43)

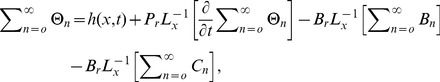
(44)


For 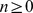 the Adomian polynomials 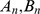 and 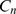 from Eqs.(43,44) are defined as
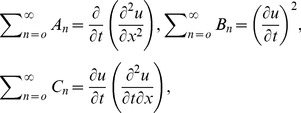
(45)


In Components form Eqs. (43,44) are derived as:
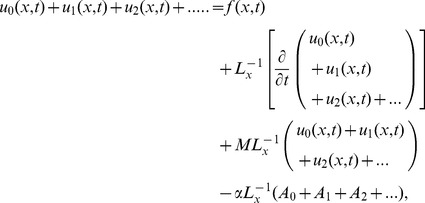
(46)

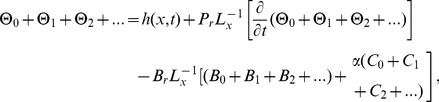
(47)


The components of velocity and temperature distribution are obtained by comparing both sides of Eqs. (46,47):

**Components of the Lift Problem up to Second Order are**:
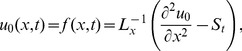
(48)

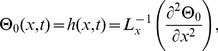
(49)

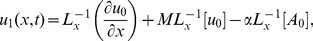
(50)

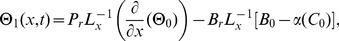
(51)

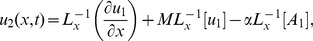
(52)

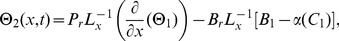
(53)


Making use of boundary conditions from Eqs.(21,22) in Eqs.(48–53) the zero, first and second components solution are obtained as:
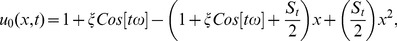
(54)

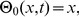
(55)

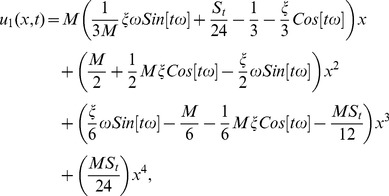
(56)

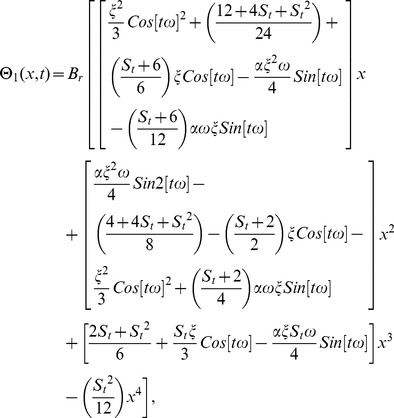
(57)

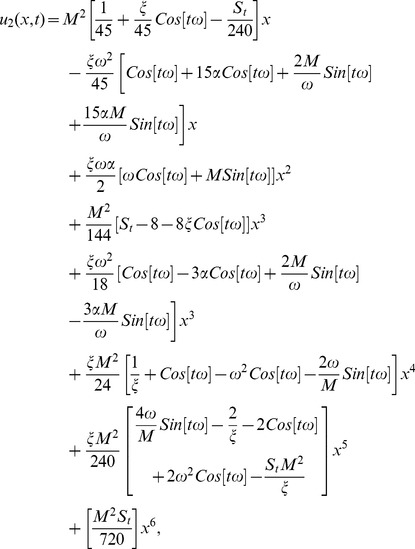
(58)


The second term solution for temperature distribution is too bulky, therefore, only graphical representations up to second order are given.

The series solution of velocity distribution up to the second component is as:
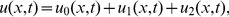
(59)


Inserting components solutions from Eqs. (54,56,58), in the series solution (59), we have:
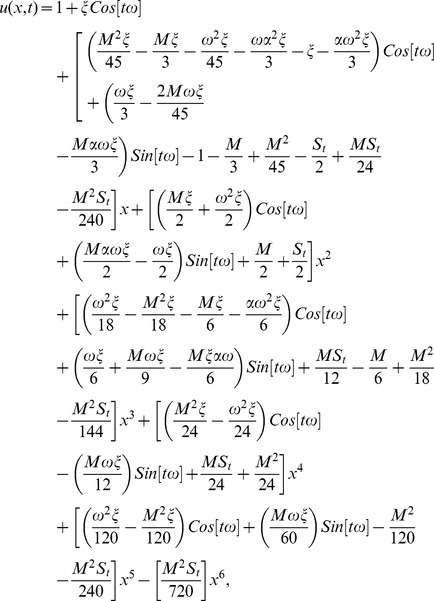
(60)


### The OHAM Solution of Lifting Problem

We construct a homotopy for Eqs. (16, 17) from the standard form of OHAM in Eq (34).

According to aforementioned discussion, the zero, first and second components problems are:
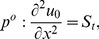
(61)

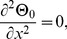
(62)

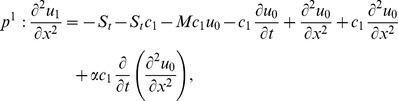
(63)

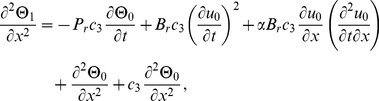
(64)

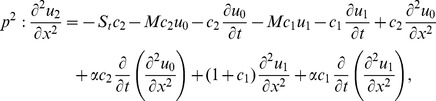
(65)

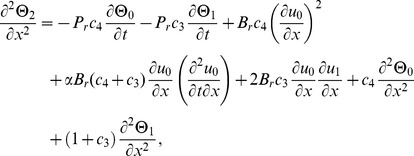
(66)


Solving Eqs. (61–66) for zero, first and second components of velocity and temperature profiles by using the corresponding boundary conditions given in Eqs. (21,22) respectively. 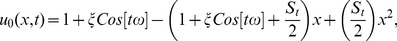
(67)

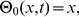
(68)

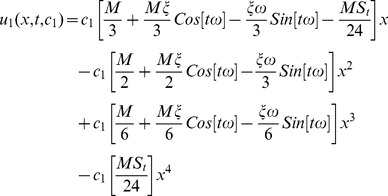
(69)

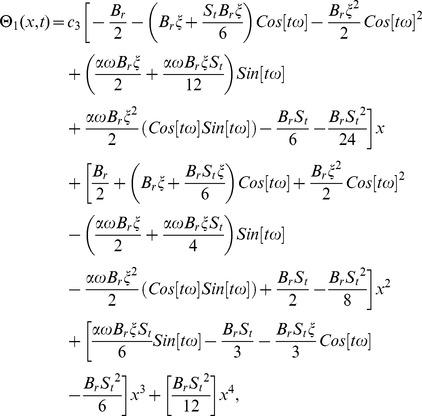
(70)


The second term solution for velocity and temperature profiles are too long, therefore, only graphical representations up to second order are given.

The arbitrary constants 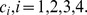 are found by using the residual:
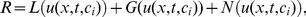
(71)


According to Eq.(36), the arbitrary constants for velocity components 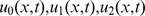 are 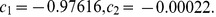


For temperature distribution, the arbitrary constants are 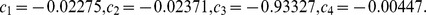


## Formulation of Drainage Problem

The geometry and assumptions of the problem are the same as in the previous problem. Consider, a film of non-Newtonian liquid drains down the vertical belt, the belt is only oscillating and the fluid drain down the belt due to gravity, so the gravity in this case is opposite to the previous case. Therefore, the Stock number is positively mentioned in Eq. (19). The coordinate system is selected same as in previous case. Assuming the flow is unsteady and laminar, fluid shear forces keeps the gravity balanced and the film thickness remains constant.

In drainage problem Eq. (19) reduced as 
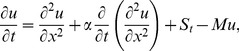
(72)


Boundary conditions for drainage problem when belt is only oscillating:
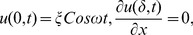
(73)


Using non-dimensional variables from Eq. (14), the boundary conditions (57) of drainage problem are reduced to:
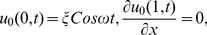
(74)


### The ADM Solution of Drainage problem

The model for drainage problem is the same as for the lift problem. The only difference in this problem is that the belt is only oscillating and due to the draining of thin film, stock number is positively mentioned in Eq. (72).

The boundary conditions for temperature distribution are the same as given in Eq. (22) but solution of these components is different. It depends on the different velocity profile of drainage and lift problems. Due to lengthy analytical calculation, solutions of temperature distribution up to first order terms are included whereas the graphical representations up to second order terms are given. Using boundary conditions (22) and (73) into Eqs. (48–53), the component solutions are obtained as:

**Components of the Lift Problem up to Second Order are:**

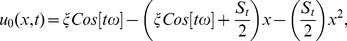
(75)

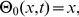
(76)

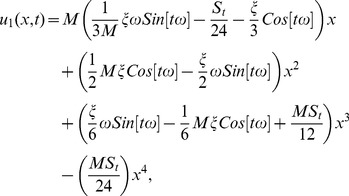
(77)

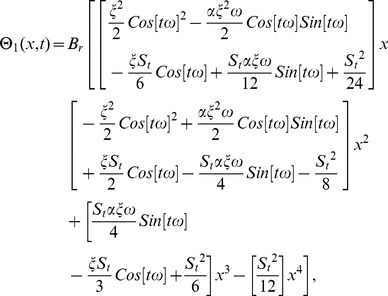
(78)

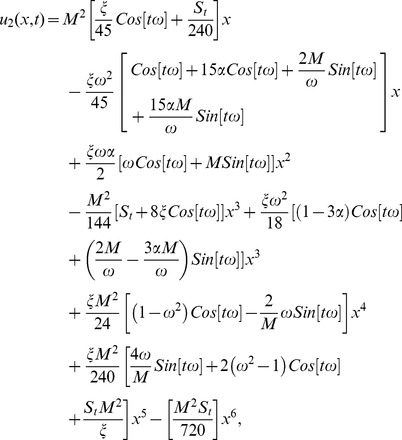
(79)


The series solution up to the second component is 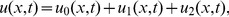
(80)inserting component solutions from Eqs. (75,77,79), in the series solution (80), we have:
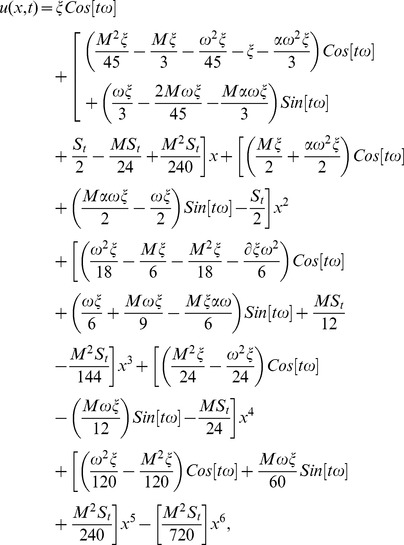
(81)


The second term solution for temperature distribution are lengthy, therefore, only graphical representations up to second order are given.

### The OHAM Solution of Drainage Problem

From the standard form of OHAM in Eq.(34), we construct a homotopy for Eqs. (72, 20).

According to the aforementioned discussion, the zero, first and second component problems are:
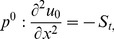
(82)

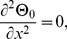
(83)

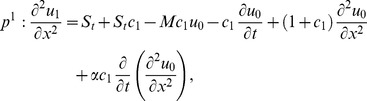
(84)

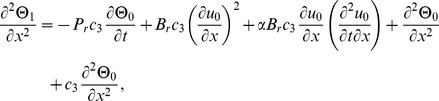
(85)

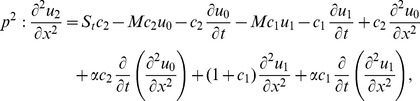
(86)

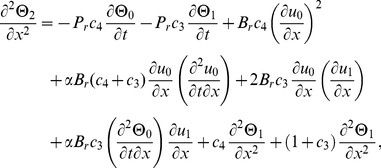
(87)


Solving Eqs. (72,20) by using the corresponding boundary conditions given in Eq. (22) and in Eq. (74). The zero component solution obtained as:
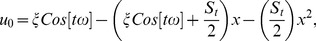
(88)

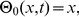
(89)

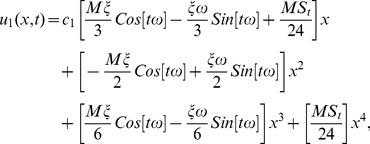
(90)

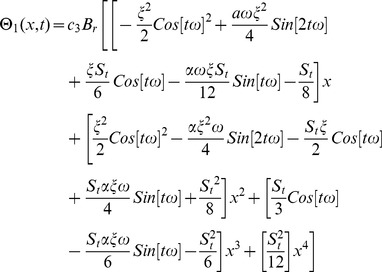
(91)


The auxiliary constants for the series solution of velocity profile and temperature distribution are respectively:
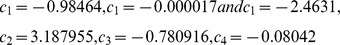



## Results and Discussion

**Figure 1:**
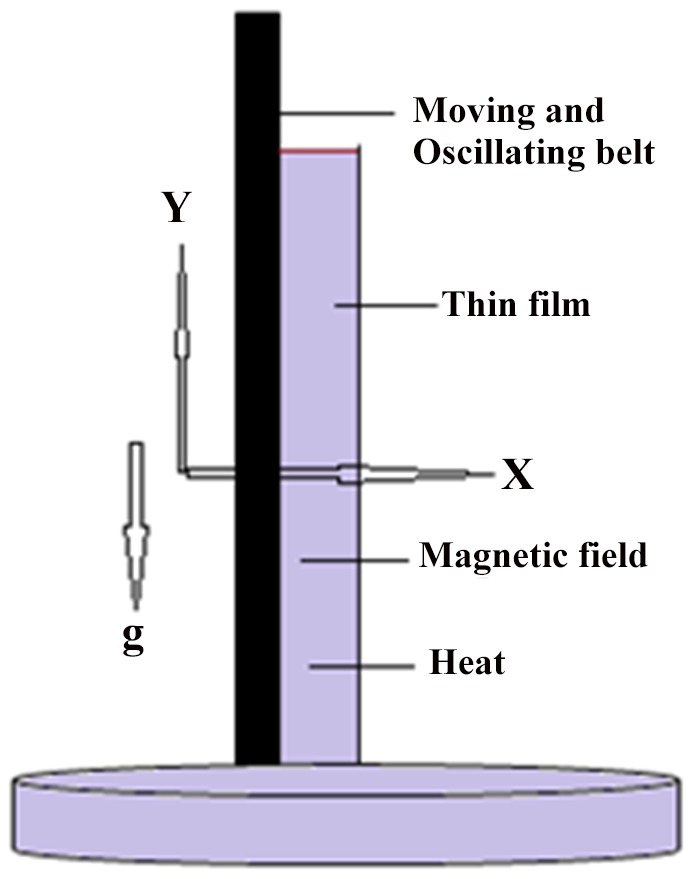
Geometry of the Lift problem.

**Figure 2:**
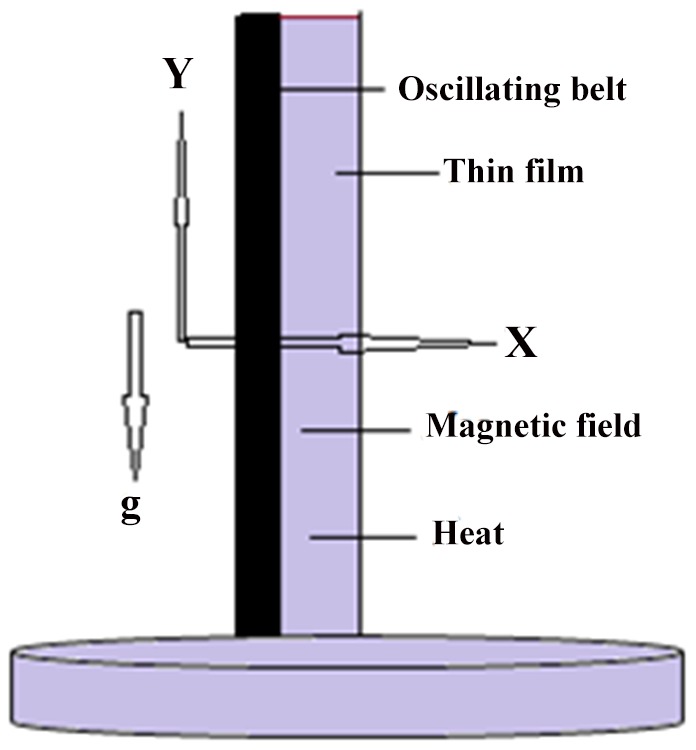
Geometry of the Drainage problem.

**Figure 3:**
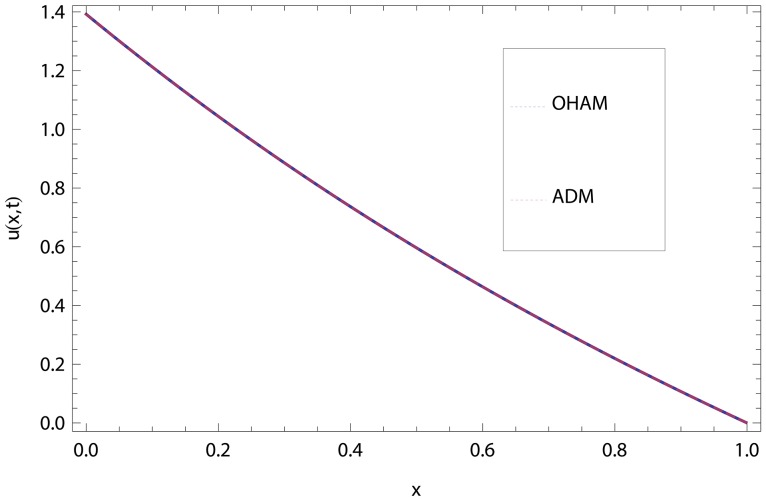
Comparison of ADM and OHAM methods for lift velocity profile. 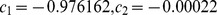
. 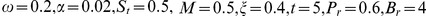.

**Figure 4:**
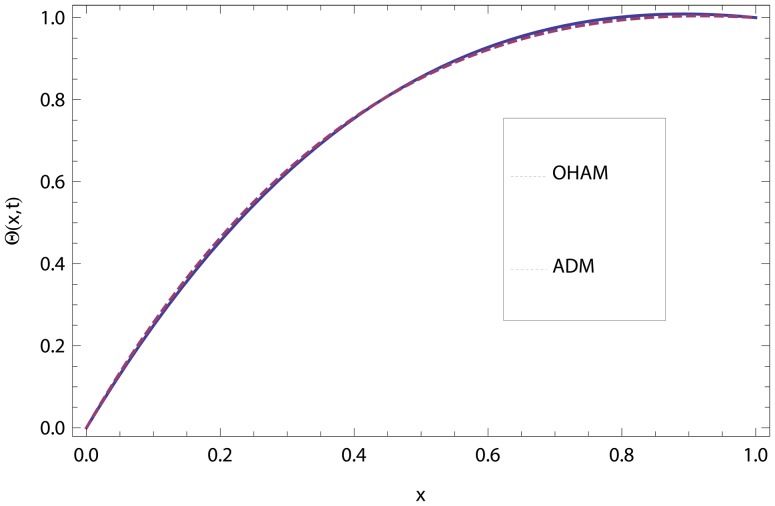
Comparison of ADM and OHAM methods for lift temperature distribution. 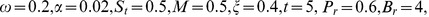
 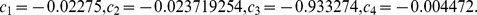

**Figure 5:**
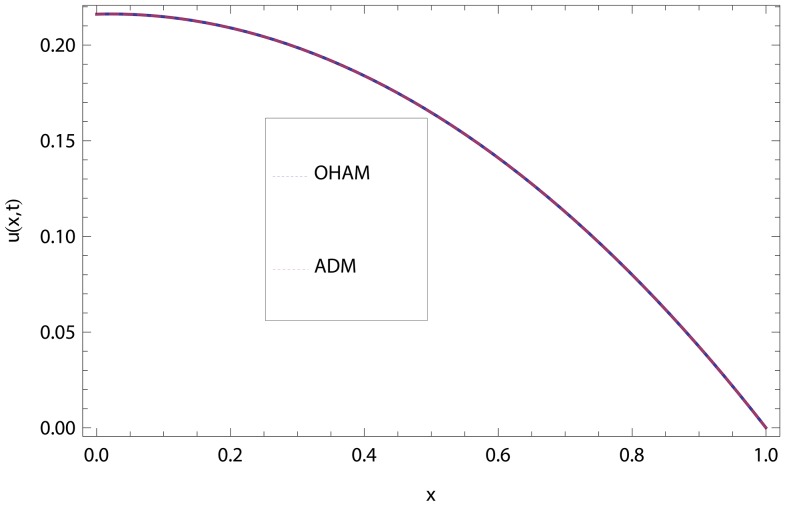
Comparison of ADM and OHAM methods for drainage velocity when 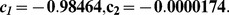. 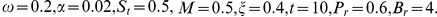

**Figure 6:**
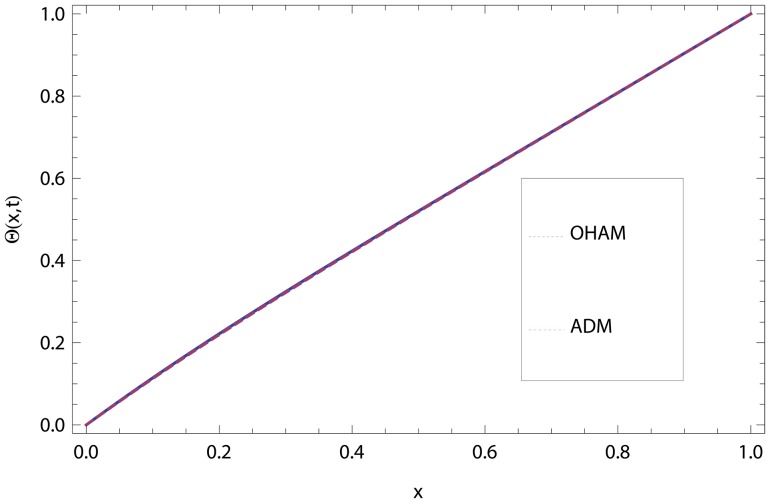
Comparison of ADM and OHAM methods for temperature distribution. 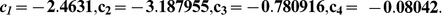
 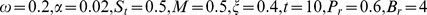.

**Figure 7:**
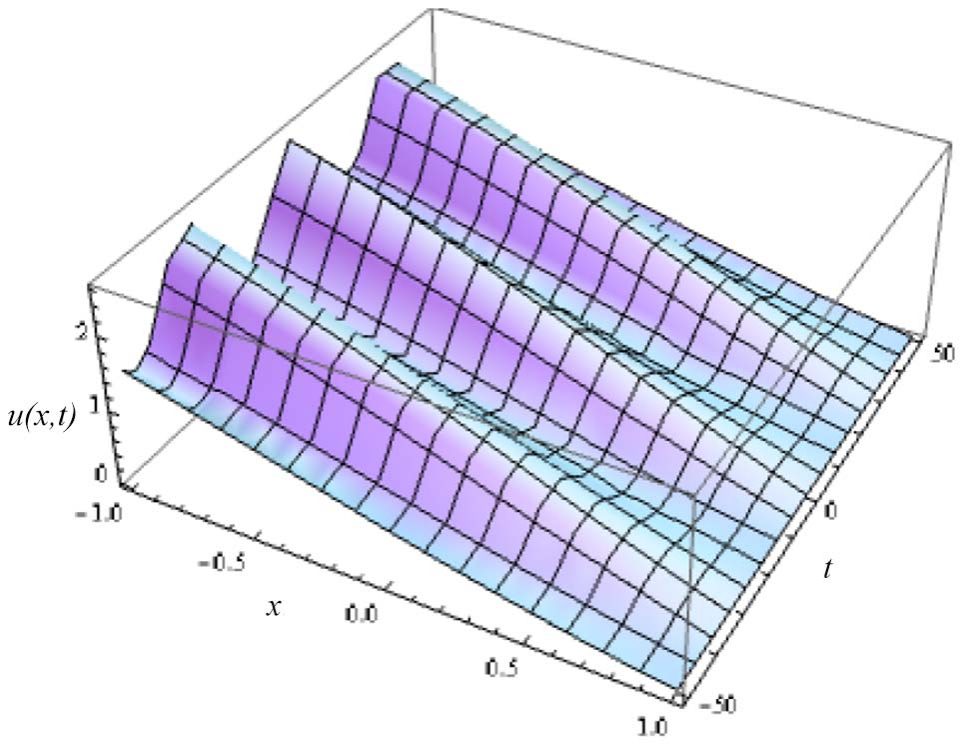
Influence of different time level on lift velocity profile.

**Figure 8:**
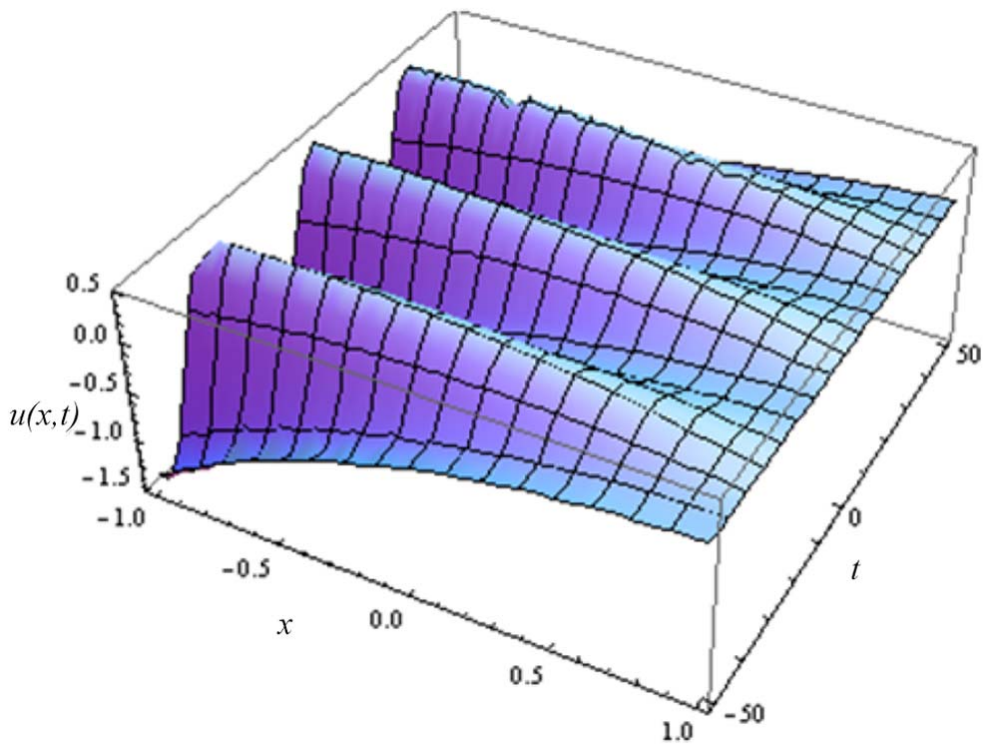
Influence of different time level on drainage velocity profile.

**Figure 9:**
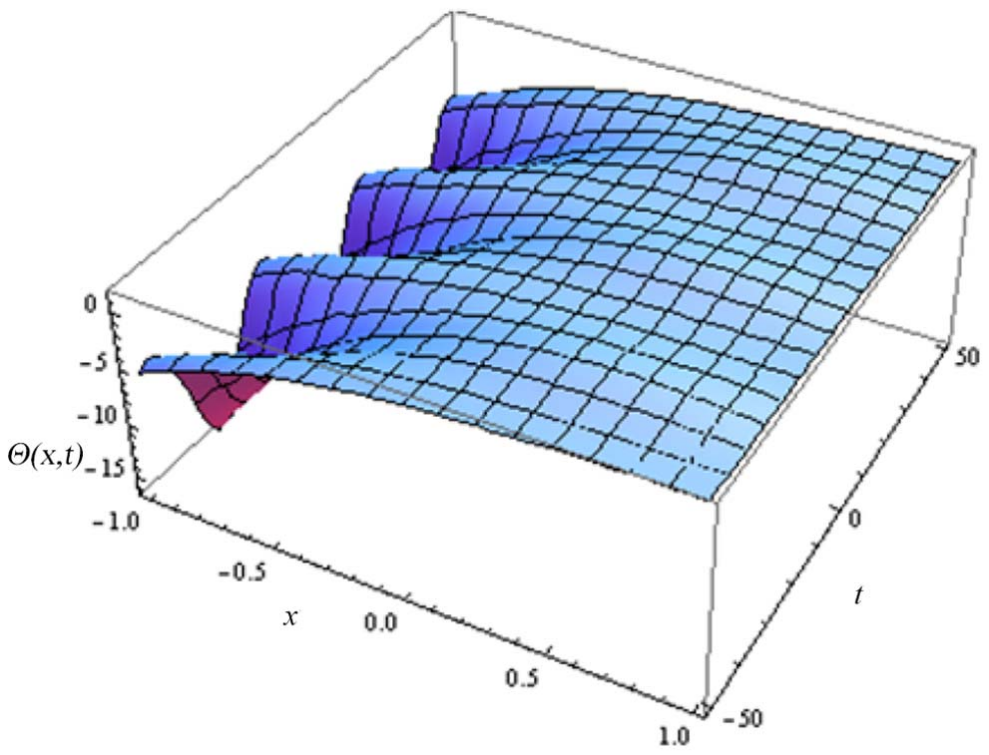
Effect of different time level on lift temperature distribution.

**Figure 10:**
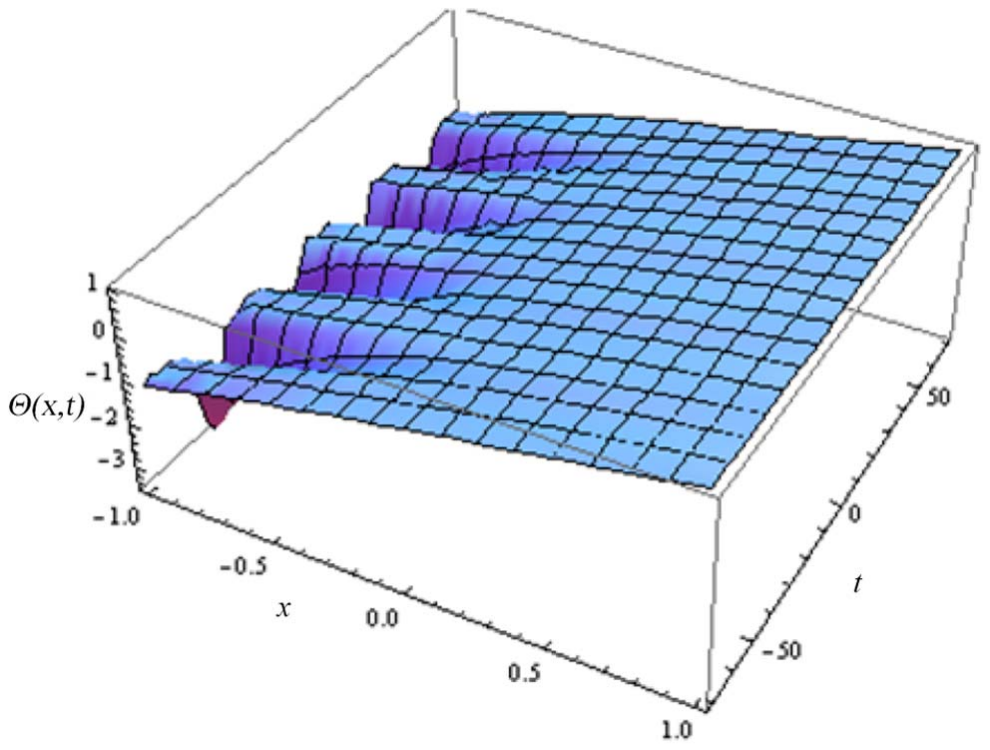
Effect of different time level on drainage temperature distribution.

**Figure 11:**
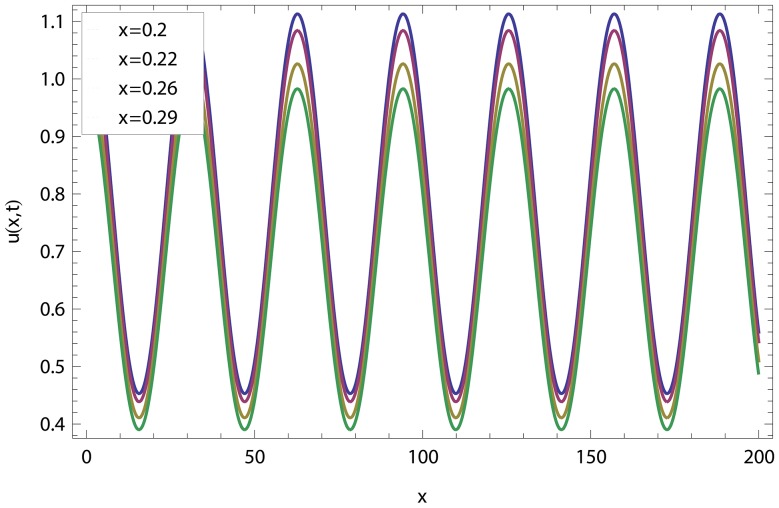
Lift velocity distribution at different time level. 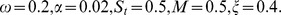

**Figure 12:**
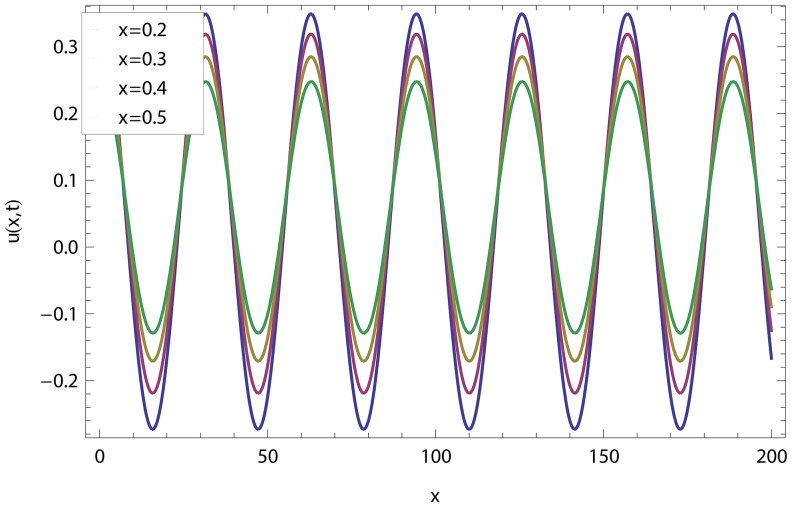
Drainage velocity distribution at different time level. 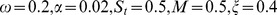
.

**Figure 13:**
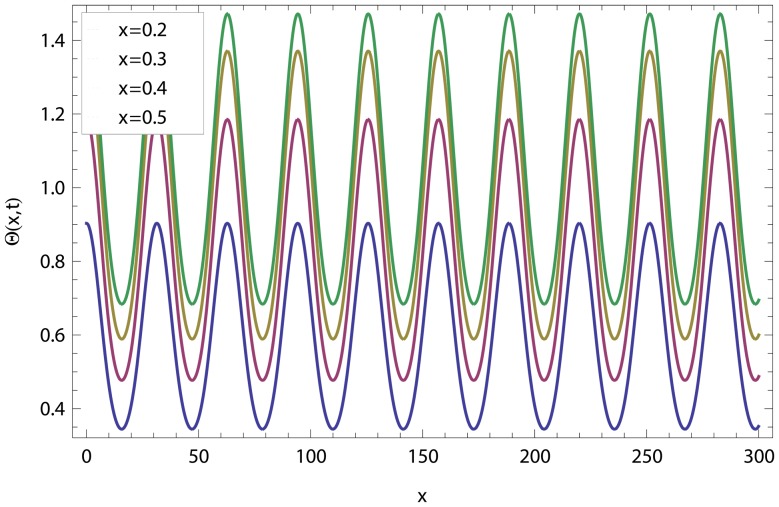
Lift temperature distribution of fluid. 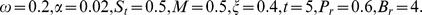

**Figure 14:**
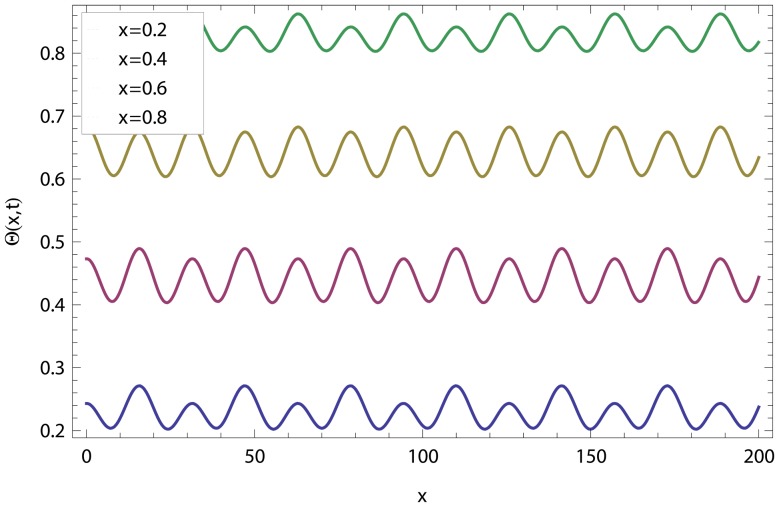
Drainage temperature distribution of fluid. 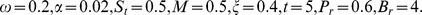

**Figure 15:**
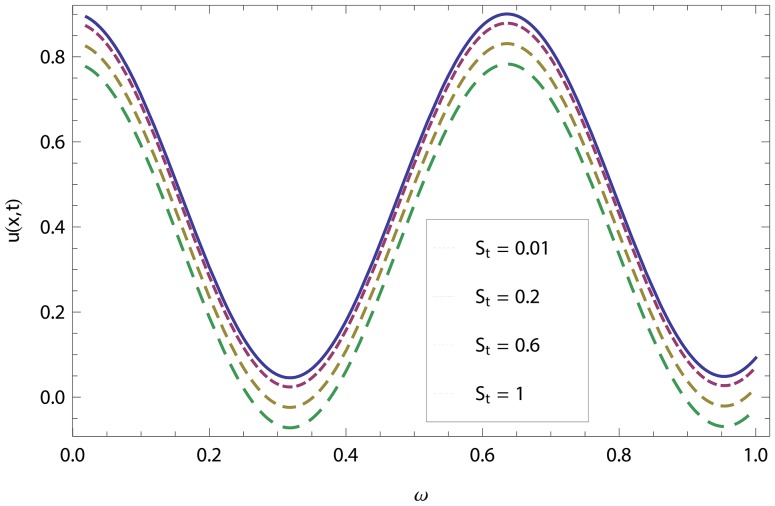
Effect of the Stock number and frequency parameter in lift velocity. 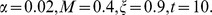

**Figure 16:**
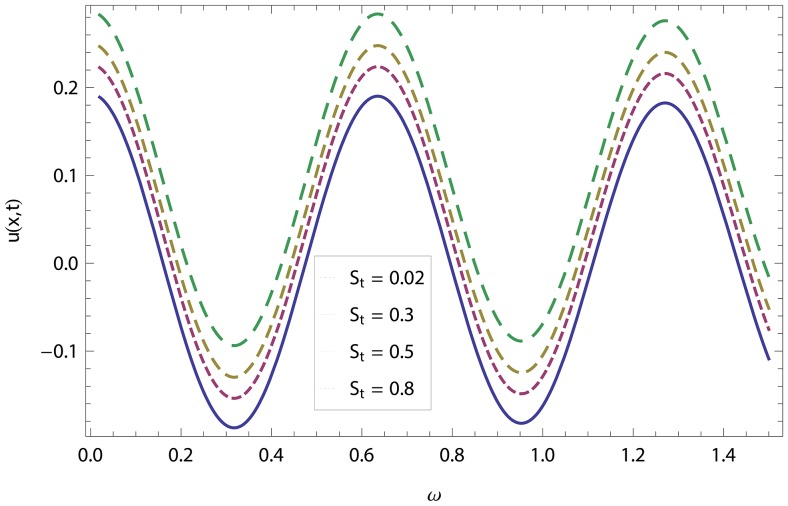
Effect of the Stock number and frequency parameter in drainage velocity. 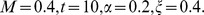

**Figure 17:**
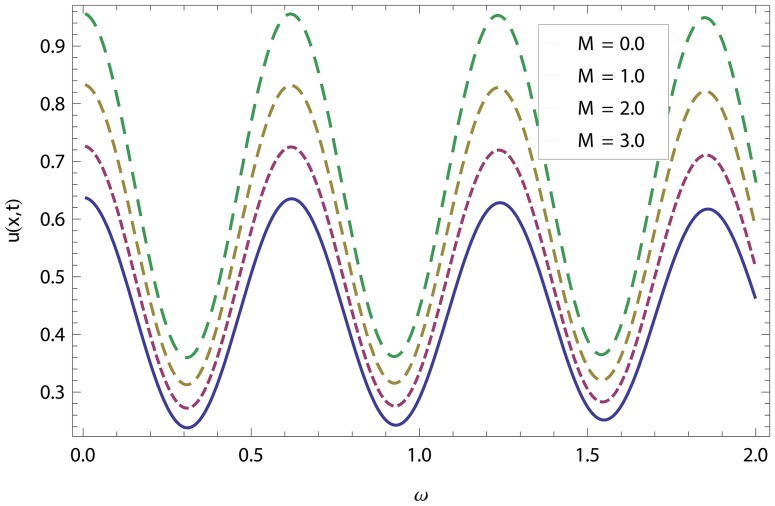
Combined effect of magnetic parameter and frequency parameter in Lift velocity. 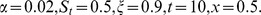

**Figure 18:**
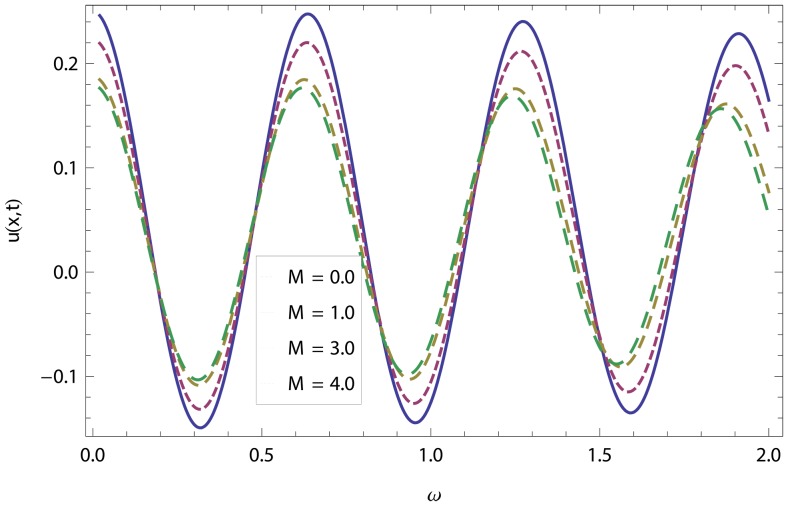
Combined effect of magnetic parameter and frequency parameter in drainage velocity. 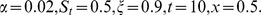

**Figure 19:**
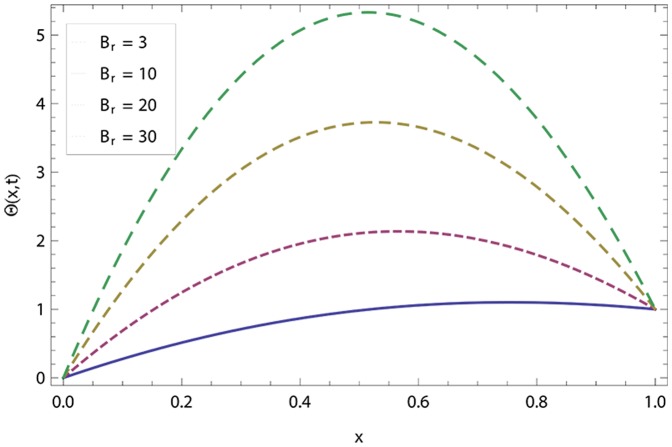
Effect of Brinkman number in lift temperature distribution. 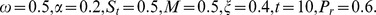

**Figure 20:**
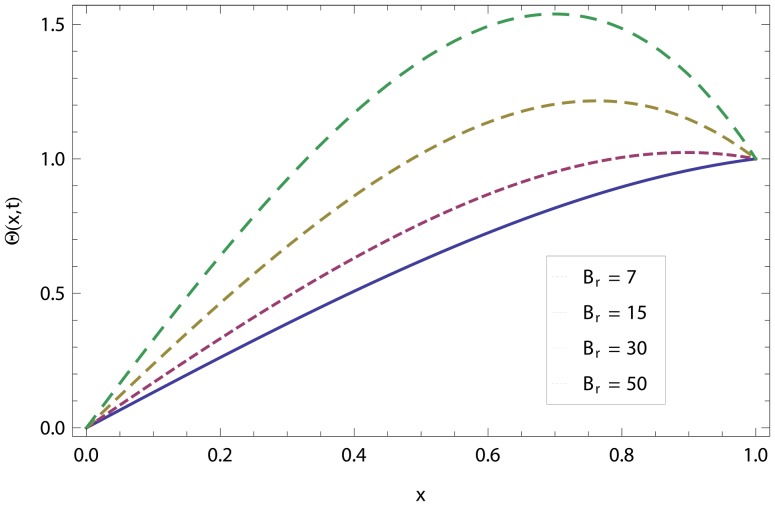
Effect of Brinkman number in drainage temperature distribution. 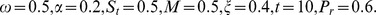

**Figure 21:**
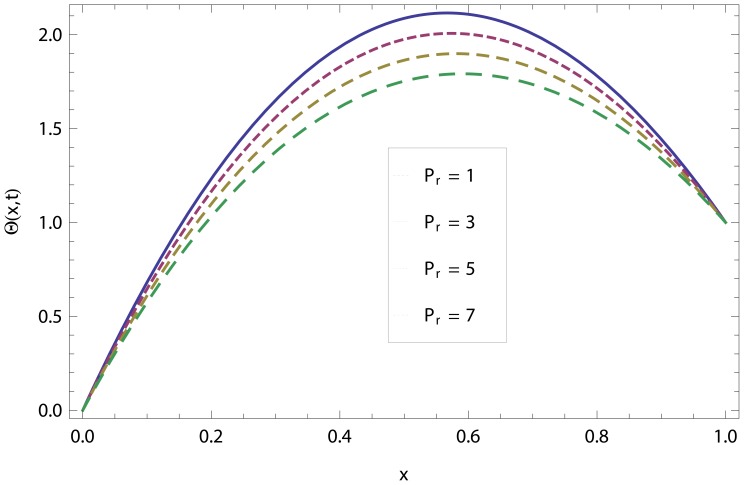
The effect of Prandtl number in lift temperature distribution. 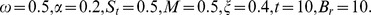

**Figure 22:**
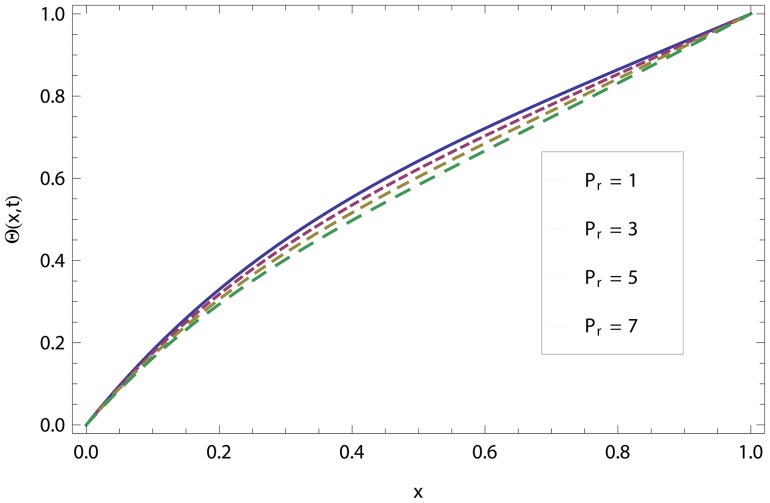
The effect of Prandtl number in drainage temperature distribution. 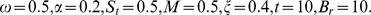

In this article, we have presented and interpreted various results for the thin film flow on a vertical oscillating belt. Figures 1 and 2 show the geometry of lift and drainage velocity profiles. The effect of non-dimensional physical parameter like Stock number 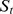, Brinkman number 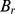, Prandtl number 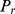 and Frequency parameter 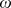 in lifting and drainage problems have been discussed in Figs. 3–22. A comparison of the ADM and OHAM solutions for velocity and temperature distribution has been shown in Figs. 3–6 for different values of physical parameters. From these Figs., we conclude that the ADM and OHAM solutions are in quite agreement. The numerical comparison of ADM and OHAM at different time level have been computed in Tables 1–4 for both lift and drainage velocity and temperature profiles respectively. It has been concluded from these tables that absolute error between ADM and OHAM decreases with decrease in time level, while it increases with increase in time level. As the flow of fluid film is subjected to the oscillation as well as translation of the belt, so the velocity and temperature distribution of the fluid film will be high at the surface of the belt comparatively to the residual domain and will decrease gradually for the fluid film away from the surface of the belt. These conclusions have been observed from Tables 1–4 and Figs 7–14. Fig. 15 shows that velocity increases in lift flow when Stock number 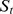 increases. Physically, it is due to friction which seems smaller near the belt and higher at the surface of the fluid. The velocity of fluid decreases with increasing Stock number in drainage problem shown in Fig 16. Physically, it is due to the fact that increasing Stock number causes the fluids' thickness and reduces its flow. When the flow of fluid is downward in oscillation, velocity increases while it decreases when the flow of fluid is upward. Variations of the magnetic parameter 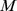 on lift and drainage velocity profiles have been studied in Figs. 17, 18. Increase in magnetic parameter increases the velocity profile in lift problem but in drainage problem, it is clear that the boundary layer thickness is reciprocal to the transverse magnetic field and velocity decreases as flow progresses towards the surface of the fluid. In lift and drainage velocity profiles, increase in non-dimensional frequency 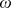 changes the direction of fluid flow frequently and steadily converges to a point on the surface of the fluid. If the belt velocity increases with oscillation, the centripetal force decreases and, as a result, velocity of fluid decreases. Figs. 19 and 20 show the effect of Brinkman number 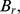 for lift and drainage temperature distribution. The temperature distribution increases as the 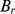 increases and becomes more trampled for higher values of 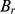. Figs. 21, 22 show the effect of Prandtl number 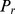 on the lift and drainage temperature distribution. In Eq. (20) Prandtl number 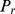 is reciprocal to other physical parameters. So increase in Prandtl number 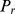 decreases the temperature distribution.

**Table 1:** Comparison of OHAM and ADM for lift velocity.

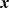	OHAM	ADM	Absolute Error
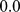	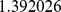	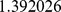	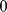
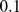	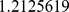	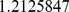	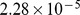
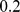	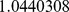	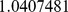	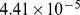
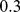	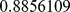	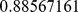	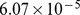
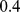	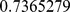	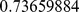	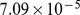
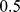	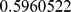	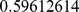	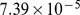
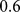	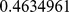	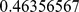	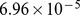
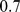	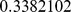	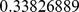	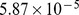
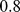	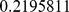	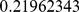	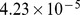
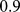	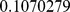	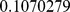	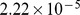
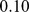	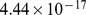	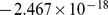	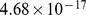

When 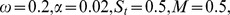 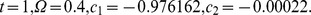 

**Table 2:** Comparison of OHAM and ADM for lift temperature distribution.

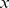	OHAM	ADM	Absolute Error
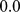	 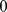	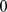	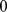
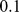	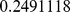	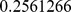	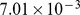
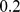	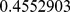	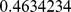	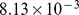
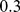	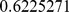	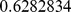	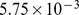
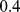	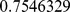	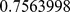	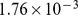
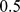	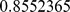	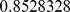	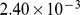
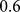	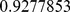	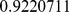	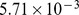
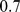	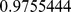	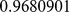	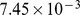
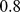	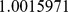	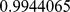	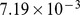
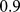	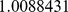	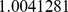	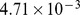
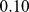	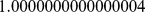	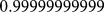	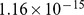

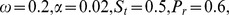
 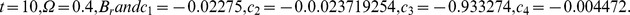

**Table 3:** Comparison of OHAM and ADM for drainage velocity profile.

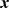	OHAM	ADM	Absolute Error
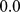	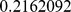	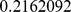	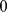
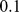	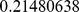	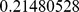	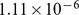
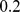	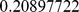	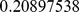	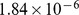
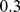	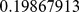	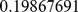	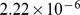
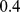	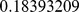	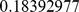	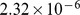
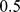	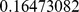	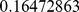	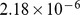
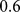	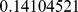	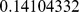	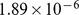
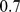	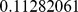	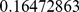	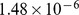
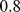	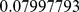	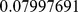	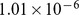
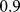	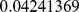	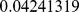	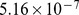
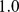	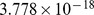	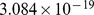	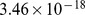

When 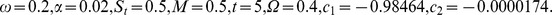

**Table 4:** Comparison of OHAM and ADM for drainage temperature distribution.

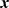	OHAM	ADM	Absolute Error
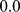	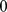	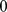	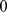
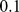	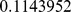	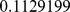	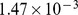
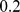	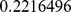	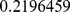	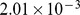
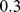	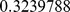	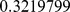	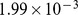
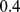	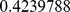	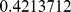	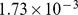
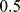	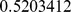	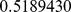	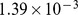
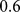	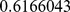	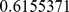	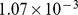
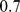	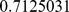	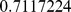	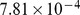
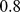	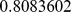	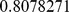	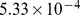
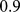	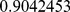	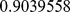	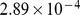
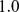	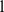	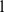	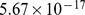

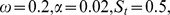
 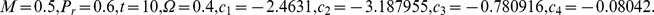

## Conclusion

In this article, we have modeled the thin film flow of unsteady second grade fluid on a vertical oscillating belt. The belt is oscillating and translating for lift velocity distribution while belt is only oscillating for drainage velocity distribution in the form of partial differential equation. Both problems have been solved analytically by ADM and OHAM. The comparison of ADM and OHAM has been derived graphically and numerically. We have concluded that the velocity and temperature distribution of the fluid film will be high at the surface of the belt comparatively to the residual domain and will decrease gradually for the fluid film away from the surface of the belt. Expression for velocity and temperature fields have been resulted and sketched. The effects of physical parameters have been sketched and discussed.
